# Decreasing age at first anal intercourse among men who have sex with men in China: a multicentre cross-sectional survey

**DOI:** 10.7448/IAS.19.1.20792

**Published:** 2016-08-10

**Authors:** Huachun Zou, Junjie Xu, Qinghai Hu, Yanqiu Yu, Gengfeng Fu, Zhe Wang, Lin Lu, Minghua Zhuang, Xi Chen, Jihua Fu, Zhenhai Zhou, Wenqing Geng, Yongjun Jiang, Hong Shang

**Affiliations:** 1 Department of Medical Statistics and Epidemiology, School of Public Health, Sun Yat-sen University, Yuexiu District, Guangzhou, P.R. China; 2 Guangdong Center for Skin Disease & STI Control, Guangzhou, Guangdong, China; 3 Kirby Institute, University of New South Wales, Sydney, Australia; 4 Key Laboratory of AIDS Immunology of National Health and Family Planning Commission, Department of Laboratory Medicine, The First Affiliated Hospital, China Medical University, Shenyang, China; 5 Collaborative Innovation Center for Diagnosis and Treatment of Infectious Diseases, Hangzhou, China; 6 Jiangsu Provincial Centers for Disease Control and Prevention, Nanjing, China; 7 He'nan Provincial Centers for Disease Control and Prevention, Zhengzhou, Zhengdong, China; 8 Yunnan Provincial Centers for Disease Control and Prevention, Kunming, China; 9 Shanghai Municipal Centers for Disease Control and Prevention, Shanghai, China; 10 Hu'nan Provincial Centers for Disease Control and Prevention, Changsha, China; 11 Shandong Provincial Centers for Disease Control and Prevention, Jinan, China; 12 National Center for AIDS/STD Control and Prevention, Chinese Center for Disease Control and Prevention, Changping District, Beijing, China

**Keywords:** MSM, AFAI, HIV, recreational drug, China, birth cohort

## Abstract

**Introduction:**

Literature on the age at first anal intercourse (AFAI) among men who have sex with men (MSM) is limited. We aimed to elucidate the evolution of AFAI and the factors associated with early AFAI, based on a large sample of MSM in China.

**Methods:**

We collected information on the demographics and sexual behaviours of MSM from seven large cities in China from 2012 to 2013. Blood samples were collected for HIV serology. AFAI was calculated for MSM born in different time periods. Linear regression models were used to explore factors associated with younger AFAI.

**Results:**

A total of 4491 MSM (median age: 27 years, median AFAI: 21 years) were recruited. Median AFAI decreased steadily from 33 years of age among MSM born from 1940 to 1959 to 18 years of age among MSM born from 1990 to 1996. Factors significantly associated with younger AFAI included more recent birth cohort, being unmarried or living with a male partner, being a student or industry worker, the gender of the first partner being male, and using Rush or Ecstasy in the past six months (*p* for all <0.05).

**Conclusions:**

AFAI among MSM in China has considerably decreased over the past few decades. The decreasing AFAI and factors associated with younger AFAI point to the necessity of early sex education and control of recreational drug use among MSM in China.

## Introduction

National representative samples in many countries have shown that both men and women are having sex earlier than before. The earlier commencement of sex implies higher numbers of sexual partners and higher sexually transmitted infection (STI) prevalence in heterosexual men and women. The Third British National Surveys of Sexual Attitudes and Lifestyles (Natsal-3) found a decline in age at first vaginal sex (men: 18–16; women: 19–16) and a climbing proportion reporting first vaginal sex before 16 years of age (men: 15.4–30.9%; women: 4.0–29.2%) in the following birth cohorts of heterosexual men and women: 65 to 74 years of age and 16 to 24 years of age [1]. The Second Australian Sexual Health and Relationships Study (ASHR2) found that for both heterosexual men and women, first vaginal sex before 16 years of age was significantly associated with a greater number of lifetime and recent sexual partners. There was also significant association with a greater likelihood of having had an STI [2].

Compared to heterosexual men and women, existing studies in developed countries found a more noticeable decrease in age at first anal intercourse (AFAI) among men who have sex with men (MSM). An online survey of 822 MSM in Australia found the median AFAI fell from 35 years of age for men born from 1944 to 1953 to 18 years of age for men born from 1984 to 1993 [3,4]. AFAI was generally younger among men who reported having more than 10 sexual partners, engaging in group sex, engaging in receptive anal intercourse (AI), or abusing drugs or alcohol, in the past 12 months [3,4]. A sample of 2200 MSM in Switzerland found the median AFAI fell from 25 years of age among men born before 1965 to 15 years of age among those born from 1985 to 1989 [5]. Data on the 574 MSM in ASHR2 found that homosexual-identifying MSM had a significantly younger mean age at first sexual experience (18.5 years) than bisexual-identifying (21.9 years) and heterosexual-identifying MSM (20.9 years) [6].

In China, MSM tended to start sexual life at an early age [7]. Six consecutive annual surveys among MSM in Guangzhou, China, found that the proportion of MSM whose AFAI was younger than 19 years of age increased from 15.5% in 2008 to 22.9% in 2013 [8]. Another study in Chongqing, China, showed that 31.2% of MSM experienced their first AI before 19 years of age [9]. Studies have demonstrated that younger MSM tended to have anal sex with older partners. Sexual risk behaviour is similar regardless of age difference between partners. Given that HIV prevalence is generally higher among older MSM, younger, HIV-negative men engaging in unprotected, receptive AI with older men have an elevated risk of HIV infection [10]. However, until now, no large sample of MSM from various parts of China has reported detailed data on AFAI. Since AFAI is on the decrease, it is necessary to understand the timing of sexual debut and its influencing factors among MSM. We aimed to show AFAI and factors associated with earlier initiation of AI in successive birth cohorts of MSM in China, based on a large cross-sectional sample of MSM from seven large cities in different parts of China.

## Methods

### Study participants

This cross-sectional study was conducted from June 2012 to June 2013 in seven large cities in China: Shanghai, Shenyang, Jinan, Changsha, Zhengzhou, Nanjing and Kunming. These cities represent different geographical locations, social and economic statuses, and the levels of the HIV epidemic across China. MSM participants were recruited using multiple approaches: advertisements on gay websites and online chat rooms, outreach to gay-gathering venues (e.g. gay bars, parks, and public bathhouses) and peer referral. Men were eligible if they were 16 years of age or older, able to provide informed consent and self-reported anal/oral sex experiences with other men. With regard to sample size, each city recruited at least 400 participants, which is the typical sample size of sentinel surveillance in a major city in China. One of the original main objectives was to estimate HIV prevalence in the seven selected study sites. A two-sided Z-test was used to estimate the needed sample size in each city. A sample size of 641 in each city was required with the assumption of statistical analysis power=90%, alpha=0.05, HIV prevalence=10% and the estimate proportion difference d_0_=0.04.

### Questionnaire interview

We collected data using a questionnaire with detailed information on (1) demographics: date of birth, location, education, marital status, ethnicity, occupation and monthly income; (2) sexual behaviours: sexual orientation, AFAI with another man, number of both male and female sexual partners, condom use in the past six months, condom use in last anal sex occurrence with a male partner, role in anal sex with a man and gender of first sex partner; and (3) other risk behaviours implying HIV/STI transmission: commercial sex in the past six months and recreational drug use experiences. To decrease the interviewer bias, interviewers in all cities received standardized training of a uniformed protocol.

### Laboratory testing

A blood sample was collected from each participant to test for HIV-1. HIV-1 antibody was tested using enzyme-linked immunosorbent assay [ELISA] (bioMerieux, Durham, NC, USA), and positive tests were confirmed by western blot test (HIV Blot 2.2 WBTM, Genelabs Diagnostics, Singapore).

### Statistical analysis

Sample characteristics were compiled using descriptive statistics. The mean and its corresponding interquartile ranges (IQRs) were used to describe age and AFAI. The proportions of men under 20 years of age and the proportions of men with AFAI under 20 years of age in each city and their corresponding 95% confidence intervals (CIs) were used to describe the proportion of teenagers and the proportion of men commencing AI as teenagers in all participants. These data were illustrated on a map of China.

The World Health Organization (WHO) defines adolescence as the period in human growth and development that occurs after childhood and before adulthood, from ages 13 to 19. We categorized MSM into less than 20 and equal to 20 years or older because of the implications to adolescent sexual health education [11].

Numerical variables such as age, AFAI and number of AI partners in the past six months were categorized and then treated as categorical variables. Proportions and their corresponding 95% CIs were used for categorical variables, such as the role in AI and condom use in the most recent AI. Variables with a *p* value of <0.1 in univariate linear regression models were entered into a multivariate linear regression to explore factors associated with younger AFAI, with a *p* value <0.05 being regarded as statistically significant. Because the number of participants with missing data in any key variables was less than 5% of the total sample size and the potential bias due to listwise deletion in the multivariate analysis is unlikely to be significant, we did not impute the missing values using methods such as the multiple imputation by chained equations. Statistical analyses were conducted using STATA 13.0 (StataCorp, Tx, USA).

### Ethical statement

The study protocol and informed consent form were reviewed and approved by the institutional review board of the First Affiliated Hospital of China Medical University. Study details were explained clearly for each participant and written informed consent was obtained before commencement of the survey.

## Results

### Participant characteristics

As shown in Table 1, a total of 4491 MSM were recruited. Median age was 27 years of age (mean 29.7 years, standard deviation (SD) 9.5 years, range 15–71 years). Two thousand nine hundred and fifty-six (65.8%) men were 30 years of age or younger. Participants’ education level was quite high: 53.0% had college education or higher compared to the national level of 8.7%. Most men self-identified as being either homosexual (59.2%) or bisexual (27.1%). Four hundred and sixty-six (10.4%) were from Zhengzhou in central China, 632 (14.1%) from Kunming in southwestern China, 662 (14.7%) from Shenyang in northeastern China, 688 (15.3%) from Changsha in mid-southern China, 675 (15.0%) from Jinan in northern China, 592 (13.2%) from Nanjing and 776 (17.3%) from Shanghai in eastern China.

**Table 1:** Mean age at first anal intercourse according to demographic characteristics among MSM in China

			Univariate regression		Multivariate regression	
				
	*n* (%)	Mean AFAI	Beta coefficient	*p*	Adjusted beta coefficient	*p*^a^
Age cohort						
1940–1959 (53–72 years)	151 (3.4)	33.2	14.78	<0.001	12.79	<0.001
1960–1969 (43–53 years)	352 (7.8)	28.7	10.23	<0.001	8.18	<0.001
1970–1979 (33–43 years)	787 (17.5)	25.1	6.55	<0.001	4.95	<0.001
1980–1989 (23–33 years)	2276 (50.7)	21.1	2.61		2.31	<0.001
1990–1996 (16–23 years)	925 (20.6)	18.5	Ref		Ref	
Education						
Secondary or below	2113 (47.0)	22.9	Ref		Ref	
Postsecondary	2378 (53.0)	21.7	−1.16	<0.001	0.07	0.677
Sexual orientation						
Homosexual	2658 (59.2)	21.7	Ref		Ref	
Bisexual	1218 (27.1)	23.7	1.49	<0.001	0.99	<0.001
Heterosexual/unidentified	615 (13.7)	22.2	0.48	0.089	0.76	0.286
Marital status						
Unmarried	3133 (69.8)	20.6	−6.46	<0.001	−1.17	<0.001
Living with male	167 (3.7)	20.6	−6.49	<0.001	−2.04	<0.001
Marriage of convenience^b^	58 (1.3)	23.5	−3.60	<0.001	−1.04	0.102
Divorced or widowed	230 (5.1)	26.7	−0.42	0.319	−0.54	0.121
Married/living with female	903 (20.1)	27.1	Ref		Ref	
Ethnicity						
Han	4191 (93.3)	22.3	Ref			
Minorities	300 (6.7)	21.8	−0.49	0.191		
Occupation						
Students	565 (12.6)	19.1	−5.21	<0.001	−1.06	0.012
Service industry workers	1083 (24.1)	21.5	−2.78	<0.001	−0.89	0.001
Government staff	436 (9.7)	23.0	−1.31	0.001	−0.41	0.201
Factory workers	495 (11.0)	24.3	Ref		Ref	
Other occupation	1638 (36.5)	23.1	−1.22	<0.001	−0.57	0.120
Unemployed	274 (6.1)	22.6	−1.73	<0.001	−0.42	0.315
Monthly income (RMB)						
No income	768 (17.1)	20.3	Ref		Ref	
1–3000	2036 (45.3)	22.8	2.48	<0.001	−0.47	0.155
> 3000	1687 (37.6)	22.6	2.28	<0.001	−0.50	0.160

AFAI: age at first anal intercourse; 1 USD~=6.2R MB.

a Adjusted for source city, role in anal intercourse, gender of first partner, condom use in recent anal intercourse with a man, number of male partners in the past six months, commercial sex with either males or females in the past six months, vaginal intercourse with women in the past six months, condom use in recent vaginal intercourse with a woman, Rush or Ecstasy use, and any sexually transmitted infection history.

b To hide homosexuality in cases where being openly gay is socially unacceptable, punishable or potentially detrimental, some gay men choose marriage for convenience. A gay man and a heterosexual woman or a gay man and a gay woman marry each other to create the appearance of heterosexuality.

### Age at first anal intercourse

**Figure 1:**
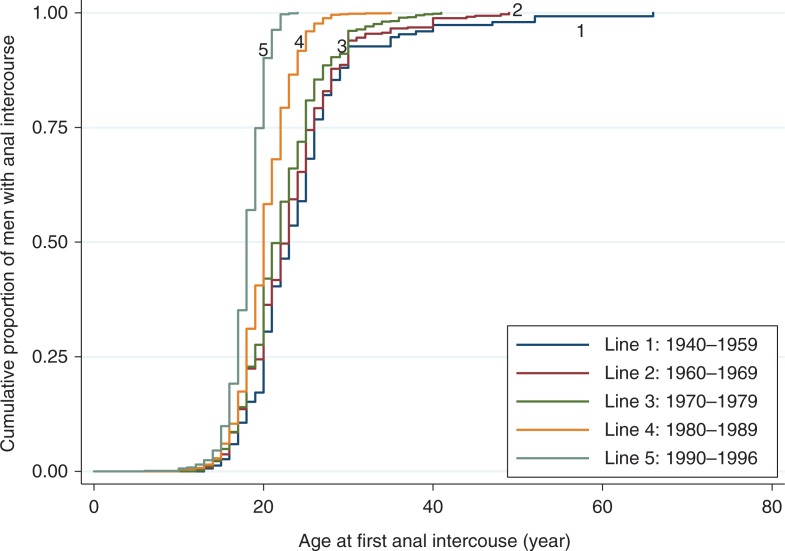
The Kaplan–Meier survival curves for age at first anal intercourse according to MSM's age cohort. The five survival curves represent MSM in different birth cohorts.

**Figure 2:**
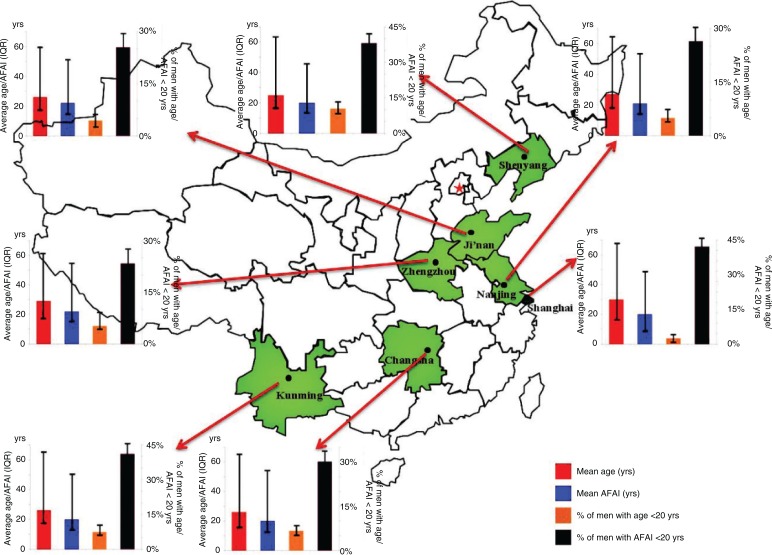
The mean age and mean AFAI among MSM in seven cities in China. AFAI: age at first anal intercourse; IQR: interquartile range; 95% CI: 95% confidence interval. Red column: mean age; vertical bar: IQR of age. Blue column: mean AFAI; vertical bar: IQR of AFAI. Orange column: percent of men with age <20 years; vertical bar: 95% CI of % of men with age <20 years. Brown column: percent of men with AFAI <20 years; vertical bar: 95% CI of % of men with AFAI <20 years. In each of the six small histograms, the left axis title is average age/AFAI (years) and the right axis title is the percentage of men with age/AFAI <20 years.

Overall, the median AFAI was 21 years of age (mean 22.3 years, SD 6.3 years) among all MSM in our study. Figure 1 displays the Kaplan–Meier survival curves of AFAI stratified by birth cohort. The proportions of MSM reporting AI in early adulthood increased as the birth cohort years became more recent. The median AFAI was substantially older for men born from 1940 to 1959 (33 years) than for those in subsequent cohorts (1960–1969: 28 years; 1970–1979: 25 years; 1980–1989: 21 years; 1990–1996: 18 years). Figure 2 demonstrates the mean age and AFAI and their corresponding IQRs, the proportions of men under 20 years of age and the proportions of men with AFAI under 20 years of age and their corresponding 95% CIs of the seven cities in China. The age of MSM was the highest in Shanghai (mean 30 years, IQR 18–67 years) and lowest in Shenyang (mean 25 years, IQR 16–64 years). The proportion of men whose AFAI was under 20 years of age was lowest in Shanghai (2.8%, 95% CI 1.8–4.3%) and highest in Shenyang (10.4%, 95% CI 8.2–13.05%). However, the proportion of MSM with AFAI under 20 years of age was the highest in Shanghai (46.8%, 95% CI 43.2–50.4%) and lowest in Zhengzhou (23.4%, 95% CI 19.6–27.5%). The mean AFAI in MSM was similar in these cities.

### Factors associated with younger AFAI

Besides the distribution of characteristics of participants, Tables 1 and 2 also demonstrate factors associated with younger AFAI. A multivariate linear regression showed the following to be more statistically significantly associated with younger AFIA: more recent birth cohort (*p<*0.001), being unmarried (*p<*0.001) or living with a male partner (*p<*0.001), being a student (*p=*0.012) or service industry worker (0.001), the gender of the first partner being male (*p<*0.001), and using Rush or Ecstasy in the past six months (*p=*0.011). All analyses above were adjusted for source city. In addition, we used a similar multivariate linear regression model with all variables except for source city included in Tables 1 and 2 for city-specific analyses. We found that more recent birth cohort and the gender of the first partner being male were associated with younger AFAI in all cities.

**Table 2:** The mean age at first anal intercourse according to risk behaviours and STI status among MSM in China

			Univariate regression		Multivariate regression	
				
	*n* (%)	Mean AFAI	Beta coefficient	*p*	Adjusted beta coefficient	*p*^a^
Role in AI						
Top	1393 (31.7)	23.4	Ref		Ref	
Bottom	976 (22.4)	21.3	−2.14	<0.001	−0.22	0.269
Versatile	2022 (45.9)	22.1	−1.36	<0.001	−0.04	0.832
Gender of the first partner						
Male	2922 (65.1)	20.2	−6.08	<0.001	−4.21	<0.001
Female	1959 (34.9)	26.2	Ref		Ref	
Condom use in recent AI with a man						
Yes	3241 (73.4)	22.1	−0.63	0.003	−0.14	0.388
No	1172 (26.6)	22.8	Ref		Ref	
Number of male partners in P6M						
0	293 (6.5)	23.0	Ref		Ref	
1–2	2453 (54.6)	22.7	−0.28	0.477	0.30	0.323
> 2	1745 (38.9)	21.6	−1.40	<0.001	−0.34	0.276
Commercial sex in P6M^b^						
Yes	459 (10.2)	20.1	−2.39	<0.001	−0.17	0.500
No	4032 (89.8)	22.5	Ref		Ref	
Vaginal intercourse with women in P6M						
Yes	832 (18.5)	24.4	2.65	<0.001	0.064	0.748
No	3659 (81.5)	21.8	Ref		Ref	
Rush or Ecstasy use in P6M						
Yes	1191 (26.5)	20.8	−2.02	<0.001	−0.42	0.011
No	3300 (73.5)	22.8	Ref		Ref	

AFAI: age at first anal intercourse; P6M: past six months; Ref: reference category; AI: anal intercourse.

a Adjusted for source city, age cohort, education, sexual orientation, marital status, occupation, and income.

b Includes selling sex to and buying sex from both men and women in the past six months.

## Discussion

This is one of the first studies that focused on AFAI and its associated sociodemographic and behavioural factors among MSM. As one of the largest MSM samples spanning a wide spectrum of birth cohorts from seven large cities in various parts of China, the study gives an in-depth understanding of the evolution of AFAI across birth cohorts and the factors associated with younger AFAI among MSM.

The average AFAI among MSM has considerably decreased over the past few decades, from 33 years of age in MSM born from 1940 to 1959 to 18 years of age in MSM born in the 1990s. It is highly likely that declines in AFAI were a result of changes in sociocultural context. Globally, homosexuality has become more accepted in different cultures over the past few decades. Beginning in 1989, when Denmark legalized same-sex marriage [12], the number of countries legalizing same-sex marriage or supporting same-sex union has been on the rise. On 26 June 2015, the United States Supreme Court ruled that same-sex couples can marry nationwide, establishing a new civil right and handing gay rights advocates a historic victory [13]. Within this global climate, the acceptance of same-sex marriage and homosexuality among general people in China is also on the rise, especially in young people [14,15]. The self-recognition of homosexual identity also increased across birth cohorts among MSM in China [16]. Other contributors included the depathologization of homosexuality from academic medical authorities and the recognition of the existence of homosexual men from the government in China. Homosexuality was removed from China's Ministry of Health's (MOH's) list of mental illnesses in 2001 [17]. In 2004, the MOH issued an official estimate of the number of male homosexuals in China [18]. With an increasingly supportive sociocultural environment, MSM are more open and willing to disclose and express their sexuality and socialize in the MSM community. Additionally, with the development of new technologies, including the Internet and smartphone-based geosocial networking applications, finding romantic and sexual partners has become considerably easier compared to a couple of decades ago [3]. This is very likely to contribute to the further decrease in AFAI among MSM.

The median AFAI of 21 years of age among MSM in our study was identical to that reported among MSM with similar median ages of other cities in China. In a sample of 541 MSM in Beijing (mean age 28.2, SD 6.9), the median AFAI was 21.0 (95% CI 21.0–22.0%) [19]. In another sample of 429 MSM in Urumqi and Beijing (median age 25), the median AFAI was 20.0 (95% CI 20.6–21.5%) [7]. In another sample of 2090 MSM in Lanzhou (median age), the median AFAI was 19.7 (SD 3.8) [20].

In April 2016, we searched in PubMed using the keywords “first AI” or “first anal sex.” We found 11 papers, only two of which provided detailed information on AFAI [4,5]. The dramatic decline in AFAI demonstrated in our study was also reflected in these two studies. In 2012, Lyons *et al*. reported AFAI among 845 MSM in Australia born from 1944 to 1993 [4]. That study found that factors associated with younger AFAI included being HIV seropositive (*p<*0.001), having more than 10 sex partners in the past 12 months (*p<*0.001), engaging in group sex (*p<*0.001), having a receptive AI (*p<*0.001) and being drug- or alcohol-affected (*p=*0.006) during their most recent sexual encounter. That study, however, recruited MSM solely from the Internet. This selection process could involve self-selection bias and information bias due to self-reported STI history. In 2009, Balthasar *et al*. reported AFAI among 2200 MSM in Switzerland (mean age 35.3 years, 65.3% aged 30 years or older) [5]. That study focused on AFAI and condom use at first AI. However, it did not report on factors associated with AFAI. By searching similar key words in the Chinese National Knowledge Infrastructure (CNKI) database, we found another study reporting AFAI among MSM in China [19]. In 2006, Li *et al*. recruited 541 MSM (mean age 28.2 years, SD 6.9) in Beijing and reported AFAI across three birth cohorts (<1970, 1970–1980, and >1980). That study found recent birth cohort (*p<*0.001) and first sex partner being male (*p<*0.001) to be significantly associated with younger AFAI [19]. Similar to Li's study, our study did not detect an association between younger AFAI and a higher number of partners or less condom use as found in Lyons’ study. It is likely that birth cohort and sociodemographic characteristics play a more important role than sexual risk profiles do in determining AFAI among MSM in China.

Our study detected association between younger AFAI and Rush (nitrite inhalants) or Ecstasy use. Studies have shown that MSM in China are increasingly using drugs, particularly Rush, that might enhance the crave and desire for sex and thus push forward AFAI [21,22]. Rush is widely available at online shops in China, normally branded as “men's perfume,” “room odorizer” or “inhalants to satisfy men's sex life.” Rush has increasingly become accepted and even became a common occurrence in the MSM population over the past few years [21]. A study including 640 MSM recruited from a voluntary counselling and testing (VCT) centre in Shenyang, China, in 2011 showed that nearly 20% had used Rush [22]. Another study from Tianjin, China, estimated that up to 80% of MSM had used Rush [21].

At early stages of one's sex life, younger MSM tend to have older partners and adopt receptive roles in anal sex [10]. The early initiation of AI and large age discrepancies were associated with risk taking: a pattern of initiation that may facilitate HIV transmission from older to younger cohorts of MSM [23]. A systematic review on global data demonstrated that MSM engaging in receptive anal sex are at higher risk for HIV infection compared to MSM engaging in insertive anal sex [24]. A Chinese study found that having first AI at age 15 or younger was significantly associated with prevalent syphilis infection (adjusted hazard ratio=9.2, 95% CI: 1.9–43.6) [25]. Similar to the decline in age at first sex in the general population, the decrease in AFAI among MSM reflects the necessity of early commencement of sexual education. Many young MSM engage in unprotected anal sex in their early sexual life, which puts them at risk for HIV/STI transmissions [10].

There were limitations to this study that need to be considered when interpreting our findings. First, due to the cross-sectional design of this observational study, temporal ambiguity did limit our ability to explore the causal relationship between potential predictors and younger AFAI. Second, convenience sampling and non-participation could have introduced some selection bias. This potential vulnerability was minimized by extensive efforts to increase representativeness and decrease non-participation. Social desirability bias was also a potential weakness owing to the collection of sensitive information, such as sociodemographic characteristics, marital status, drug use and sexual behaviours. Third, the survey was conducted in seven cities only. As a result, the study results may not represent MSM from other cities or rural areas in China. However, due to stigma and other social factors, a national representative sample of the MSM population is not likely to be achieved in the near future. Fourth, sexual debut could have taken place several years ago, which may lead to recall bias.

Until now, few MSM studies have addressed detailed information on AFAI and its associated factors. Accurate understanding of the sexual behaviours of MSM requires a large sample including MSM at different age groups. The potential influence of Internet- and smart phone-based geosocial networking applications on AFAI warrants evaluation.
